# Molecular Hydrogen as an Antioxidant and Radioprotector: Mechanistic Insights from Monte Carlo Radiation-Chemical Simulations

**DOI:** 10.3390/antiox14091054

**Published:** 2025-08-27

**Authors:** Sumaiya Akhter Ria, Jintana Meesungnoen, Jean-Paul Jay-Gerin

**Affiliations:** Department of Medical Imaging and Radiation Sciences, Faculty of Medicine and Health Sciences, Université de Sherbrooke, 3001, 12th Avenue Nord, Sherbrooke, QC J1H 5N4, Canada; sumaiya.akhter.ria@usherbrooke.ca (S.A.R.); jintana.meesungnoen@usherbrooke.ca (J.M.)

**Keywords:** water radiolysis, free radicals and reactive oxygen species (ROS), cellular oxidative damage, selective scavenging capacity, radioprotector, antioxidant, Monte Carlo track chemistry simulations, dose-rate effects

## Abstract

(1) Background: Water, comprising about 70–80% of cellular mass, is the most abundant constituent of living cells. Upon exposure to ionizing radiation, water undergoes radiolysis, generating a variety of reactive species, including free radicals and molecular products. Among these, hydroxyl radicals (^•^OH) are particularly damaging due to their very high reactivity and their capacity to induce oxidative injury to vital biomolecules such as DNA, membrane lipids, and proteins. From a radiation-chemical perspective, this study investigates the selective scavenging ability of molecular hydrogen (H_2_) toward ^•^OH radicals, with the aim of evaluating its potential as an antioxidant and radioprotective agent; (2) Methods: We employed our Monte Carlo track chemistry simulation code, IONLYS-IRT, to model the time-dependent yields of ROS in a neutral, aerated aqueous environment. The simulations included varying concentrations of dissolved H_2_ and, for comparison, cystamine—a well-known sulfur-containing radioprotector and antioxidant. Irradiation was simulated using 300 MeV protons, chosen to mimic the radiolytic effects of low linear energy transfer (LET) radiation, such as that of ^60^Co γ-rays or fast (>1 MeV) electrons; (3) Results: Our simulations quantitatively demonstrated that H_2_ selectively scavenges ^•^OH radicals. Nevertheless, its scavenging efficiency was consistently lower than that of cystamine, which produced a faster and more pronounced suppression of ^•^OH due to its higher reactivity and superior radical-quenching capacity; (4) Conclusions: Molecular hydrogen offers several unique advantages, including low toxicity, high diffusivity, selective scavenging of ^•^OH radicals, and well-documented anti-inflammatory effects. Although it is less potent than cystamine in terms of radical-scavenging efficiency, its excellent safety profile and biological compatibility position H_2_ as a promising radioprotector and antioxidant for therapeutic applications targeting radiation-induced oxidative stress and inflammation.

## 1. Introduction

The human body is composed predominantly of water, which plays a vital role in essential physiological functions such as thermoregulation, nutrient transport, waste elimination, and cellular metabolism. Upon exposure to ionizing radiation, the primary interactions occur with intracellular water molecules, leading to both ionization and electronic excitation [1,2]. Ionization generates highly unstable water radical cations (H_2_O^•+^), which undergo ultrafast decomposition—typically within ~50 femtoseconds [3]—via a pseudo-first-order proton transfer reaction with a neighboring water molecule, yielding hydroxyl radicals (^•^OH) and hydronium ions (H_3_O^+^):

H_2_O^•+^ + H_2_O → H_3_O^+^ + ^•^OH. (1)

Concurrently, excitation produces electronically excited water molecules (H_2_O*), which dissociate in the liquid phase primarily to yield ^•^OH radicals and hydrogen atoms (H^•^): H_2_O* → H^•^ + ^•^OH [4]. While excitation contributes to radical formation, its overall impact remains relatively minor compared to the predominant ionization-driven processes.

Hydroxyl radicals are among the most powerful oxidizing species and react indiscriminately with nucleic acids, lipids, and proteins. They are estimated to account for about two-thirds of the DNA damage induced by X-rays or γ-rays in mammalian cells [5,6]. For instance, in irradiated biological environments, ^•^OH can readily abstract hydrogen atoms from neighboring bio-organic molecules (RH):

^•^OH + RH → R^•^ + H_2_O (2)

with a rate constant (*k*) typically in the range of 10^8^ to 10^9^ M^−1^ s^−1^ [7]. Under oxidative stress conditions, the resulting carbon-centered radicals (R^•^) rapidly react with molecular oxygen (O_2_) to form peroxyl radicals (ROO^•^):

R^•^ + O_2_ → ROO^•^ (3)

at or near the diffusion-controlled limit (*k*~2 × 10^9^ M^−1^ s^−1^). These peroxyl radicals are often more potent oxidants than their precursor radicals. Once formed, they can irreversibly modify the parent molecules—a process known as the “fixation” of damage by molecular oxygen, in which O_2_ renders the initial radiation-induced changes permanent [7,8].

Because of their high reactivity, ^•^OH radicals, peroxyl radicals, and other species collectively termed “reactive oxygen species” (ROS) can initiate oxidative chain reactions, notably lipid peroxidation and DNA oxidation. At physiological concentrations, i.e., at low levels, ROS also serve essential functions in cell signaling and immune defense (see, e.g., [9]). However, when their levels exceed the buffering capacity of endogenous antioxidants—as can occur in radiotherapy—an imbalance arises, leading to oxidative stress. This condition causes extensive and often irreversible damage to key cellular macromolecules—including DNA, proteins, and membrane lipids—ultimately compromising cell viability and disrupting normal physiological functions [9,10,11]. Persistent oxidative stress has also been implicated in the pathogenesis of many diseases, such as cancer, cardiovascular diseases, neurodegenerative disorders including Alzheimer’s and Parkinson’s, and even in the biological aging process [10,12,13].

Another prominent example of the biological implications of water radiolysis is nanomaterial-assisted radiotherapy [14], where high-*Z* or catalytic nanoparticles can locally amplify the radiolytic production of ^•^OH and other ROS, thereby enhancing DNA damage and tumor cell killing. In this context, our quantitative analysis of ^•^OH scavenging by molecular hydrogen provides mechanistic insight into how antioxidants and radioprotectors may modulate these processes, with potential implications for optimizing therapeutic outcomes.

To mitigate ROS-induced damage—whether generated under normal physiological conditions or triggered by exposure to ionizing radiation, cells rely on a complex network of endogenous antioxidant defenses. These include small molecules such as glutathione, ascorbic acid (vitamin C), and α-tocopherol (vitamin E), along with enzymatic systems like superoxide dismutase (SOD), catalase, and glutathione peroxidase—a selenium-containing enzyme. Together, these components act synergistically to detoxify ROS, either by donating electrons or hydrogen atoms or by catalyzing their conversion into less reactive species.

However, when ROS production exceeds the neutralizing capacity of these intrinsic antioxidant defenses, supplementation with exogenous antioxidants may become necessary to restore redox balance. The cytoprotective efficacy of an antioxidant is largely determined by its radical-scavenging capacity—that is, its ability to neutralize free radicals before they can inflict oxidative damage on vital cellular targets [10,15].

From a radiation chemistry perspective, many antioxidants act as effective chemical (i.e., non-biological) radioprotectors. Their protective effect arises from their ability to scavenge the highly reactive species formed during the radiolysis of intracellular water [1,2,16], including ^•^OH radicals, hydrated electrons (e^−^_aq_), H^•^ atoms, hydrogen peroxide (H_2_O_2_), and—under aerated conditions—peroxyl species such as hydroperoxyl (HO_2_^•^) and superoxide anion (O_2_^•−^) radicals ([17]; see infra). This ROS-scavenging capacity constitutes a fundamental mechanism of radioprotection and closely mirrors the classical antioxidant role in counteracting oxidative damage. In this context, antioxidant activity represents a principal pathway through which radioprotective agents exert their cytoprotective effects [18,19,20,21,22,23,24].

Notably, no enzymatic system is known to be capable of detoxifying ^•^OH radicals, which are widely considered the most cytotoxic of all ROS. Consequently, the direct scavenging of ^•^OH constitutes a crucial line of antioxidant defense. In this context, molecular hydrogen (dihydrogen, H_2_) has been shown to act as a selective, non-toxic antioxidant—or radioprotector—by specifically reacting with hydroxyl radicals [25,26,27,28,29,30,31,32,33,34], as illustrated by the following exothermic reaction [2,10,35]:

^•^OH + H_2_ → H^•^ + H_2_O. (4)

This reaction proceeds with a rate constant of 4 × 10^7^ M^−1^ s^−1^ in water at 25 °C [36]. In an aerobic cellular environment, the hydrogen atom produced is rapidly scavenged by oxygen, forming the HO_2_^•^ radical:

H^•^ + O_2_ → HO_2_^•^, *k* = 1.3 × 10^10^ M^−^^1^ s^−^^1^. (5)

At physiological pH, HO_2_^•^ exists in its deprotonated form, O_2_^•−^ [17] (see infra), which subsequently undergoes dismutation catalyzed by SOD (see, e.g., [37] and cited references):

O_2_^•−^ + O_2_^•−^ + 2H^+^ → O_2_ + H_2_O_2_, *k*~4 × 10^9^ M^−^^1^ s^−^^1^. (6)

Taken together, this sequence of reactions (4)–(6) prevents hydroxyl radical-associated biomolecular damage by redirecting radical chemistry toward less reactive species that are more effectively neutralized by endogenous antioxidant systems.

In addition to its reactivity with ^•^OH, H_2_ has also been implicated in mitigating the effects of peroxynitrite (ONOO^−^) or yet its conjugate acid, peroxynitrous acid (ONOOH; p*K*_a_ ≈ 6.8 at 37 °C)—two highly cytotoxic “reactive nitrogen species” (RNS) capable of damaging a broad range of biological targets [38,39]. ONOO^−^ is generated under physiological conditions via the rapid, diffusion-controlled reaction between nitric oxide (^•^NO, also known as nitrogen monoxide)—a lipid-soluble, chain-breaking free radical produced endogenously in most mammalian cells—and superoxide anion radicals [40,41]:

^•^NO + O_2_^•−^ → ONOO^−^. (7)

This reaction proceeds with a rate constant of 1.9 × 10^10^ M^−1^ s^−1^, which greatly exceeds that of the competing enzymatic dismutation of O_2_^•−^ catalyzed by SOD at physiological pH [see reaction (6)].

To date, however, the direct reaction between H_2_ and peroxynitrite:

ONOO^−^ + H_2_ → NO_2_^−^ (or less reactive species) + H_2_O (8)

remains mechanistically unclear and has not been kinetically characterized. Nevertheless, experimental studies have shown that dissolved H_2_ can attenuate oxidative damage associated with ONOO^−^ in various biological systems [27]. Reaction (8), however, is not believed to be diffusion-controlled and is presumed to proceed considerably more slowly than reaction (4). While the mechanistic details of H_2_–ONOO^−^ interactions remain to be clarified, the available evidence suggests that H_2_ may exert a broader antioxidant and radioprotector effect—extending beyond oxygen-centered radicals to include nitrogen-derived oxidants produced during radiolytic or inflammatory stress.

Molecular hydrogen reacts with hydroxyl radicals more slowly than conventional radioprotectors or antioxidants such as cystamine—a sulfur-containing compound with higher chemical reactivity that rapidly scavenges free radicals [24,42]. This comparatively lower reactivity of H_2_ stems from its stable, non-polar molecular structure. Nevertheless, H_2_ is often regarded as a superior antioxidant to cystamine due to several distinct advantages. It selectively neutralizes the most cytotoxic species—particularly ^•^OH and peroxynitrite (ONOO^−^)—while exhibiting excellent biocompatibility and inherent non-toxicity [27,31]. In addition, its small molecular size and high diffusibility allow for rapid diffusion across biological membranes, including the blood–brain barrier [43,44,45]. As a result, H_2_ can exert antioxidant effects within the central nervous system, where it may help prevent or delay the onset of neurodegenerative changes [46,47].

Furthermore, several convenient and effective in vivo delivery methods for H_2_ have been developed. These include, as reviewed in detail in [31,33,34], inhalation of H_2_ gas, oral ingestion of H_2_-dissolved water (H_2_-water), intravenous or intraperitoneal injection of H_2_-dissolved saline (H_2_-saline), topical application via H_2_-enriched water baths, and ocular administration through H_2_-saline eye drops. Beyond its antioxidant and cytoprotective properties, the safety, ease of use, and versatility of H_2_ delivery significantly enhance its appeal as a preventive and therapeutic agent, making it more acceptable and accessible than many conventional antioxidants or radioprotectors.

The objective of this study was to investigate the time-dependent evolution of radiolytic species during the radiolysis of aerated and deaerated water at room temperature using 300 MeV protons—selected to mimic conventional low linear energy transfer (LET) radiation such as ^60^Co γ rays or fast electrons—in both the presence and absence of added H_2_. Using Monte Carlo track chemistry simulations ([4,42,48] and references therein), we specifically examined whether H_2_ acts as a true antioxidant by selectively reducing the yield of hydroxyl radicals in neutral water, with and without dissolved O_2_. We also explored the effect of varying H_2_ concentrations (0–10 mM) on ^•^OH yields, extending beyond the commonly cited experimental or clinical range of ~0.3 mM up to the solubility limit of ~0.78 mM (~1.57 mg/L or 1.57 ppm) under normal atmospheric pressure for H_2_-saturated drinking water (see, e.g., [27,29,49,50]). Finally, we assessed the antioxidant efficiency of cystamine in scavenging ^•^OH radicals under equivalent concentration conditions and compared its performance with that of H_2_.

All simulations were performed at 25 °C. Radiation chemical yields are reported in molecules per 100 eV of absorbed energy, using *g*(X) for primary yields and *G*(X) for experimental values. To align with the System International (SI) of Units (mol/J), we use the conversion: 1 molecule/100 eV ≈ 0.10364 μmol/J [1,2].

## 2. Materials and Methods

### 2.1. Low-LET Radiolysis of Pure Deaerated and Aerated Water: Time Scale of Events, Formation of Radical and Molecular Products, and Monte Carlo Track Chemistry Modeling

Water radiolysis is a complex multistage process that begins with the initial energy deposition by ionizing radiation—referred to as the physical stage. This is followed by the physicochemical and spatially nonhomogeneous chemical stages, during which reactive species are formed and evolve within expanding “spurs” along the track of the radiation [1,51,52]. Under low-LET irradiation, these processes typically unfold over time scales of up to ~0.2 μs after the initial ionization event at 25 °C [53]. Beyond this point—once the spurs have dissipated—the remaining radiolytic products are generally considered to be homogeneously distributed throughout the bulk solution. A detailed account of these stages has recently been provided in [1].

In the radiolysis of pure, deaerated (air-free) water by ^60^Co γ rays, fast electrons, or several hundred MeV protons (LET ~0.3 keV/μm), the main reactive species present at homogeneity include the “radical” products e^−^_aq_, H^•^, and ^•^OH, as well as the “molecular” products H_2_ and H_2_O_2_. The yields of these species at this point in time, traditionally referred to as primary or “escape” yields, are as follows [1]:

*g*(e^−^_aq_) = 2.65, *g*(H^•^) = 0.60, *g*(H_2_) = 0.45, *g*(^•^OH) = 2.80, *g*(H_2_O_2_) = 0.68. (9)

It is noteworthy that a large portion of H_2_ is produced during the early physicochemical stage of radiolysis, rather than through intra-spur chemical reactions [54,55,56]. Moreover, as a gaseous product, radiolytically formed H_2_ tends to escape readily from the solution. However, when retained, it can react with oxidizing ^•^OH radicals through reaction (4). This scenario is particularly relevant to the present study, which investigates the radiolysis of hydrogen-rich water, i.e., water containing added H_2_.

The Monte Carlo track chemistry code IONLYS-IRT, developed in our laboratory, was used to simulate the radiolysis of aerated and deaerated water, with or without H_2_ and cystamine, at 25 °C under 300 MeV proton irradiation. As noted above, these protons mimic the low-LET limit of ^60^Co γ rays or fast electrons. The code has undergone extensive validation against experimental data obtained under diverse conditions—spanning variations in pH, temperature, dose rate, LET, and solute composition—from multiple laboratories worldwide. This comprehensive benchmarking underscores its robustness and reliability across a wide range of irradiation conditions. A detailed description of the code is provided elsewhere (see, e.g., [4] and references therein).

Briefly, our event-by-event IONLYS program [57] simulates all events occurring during the early physical and physicochemical stages of radiation action, up to ~1 picosecond. The resulting complex and highly nonhomogeneous spatial distribution of species—e^−^_aq_, H_3_O^+^, OH^−^, H^•^, H_2_, ^•^OH, H_2_O_2_, HO_2_^•^/O_2_^•−^, ^•^O^•^(^3^*P*), O^•−^, and others—serves as the input for the subsequent chemical stage of the simulation. In the third stage (>1 ps), radiolytic species diffuse randomly according to their diffusion coefficients and react either with each other or with dissolved solutes—such as oxygen (in aerated solutions), H_2_, or cystamine, as in the cases studied here—present during irradiation. This stage is modeled using our IRT program [58], which is based on the “independent reaction times” (IRT) method [59,60,61], a computationally efficient stochastic approach that avoids tracking individual particle trajectories. The accuracy of this method has been validated through comparison with full random flight (or “step-by-step”) Monte Carlo simulations over a wide range of irradiation conditions [62,63]. Notably, the IRT code can also be used effectively to model homogeneous chemistry at later times, when radiation tracks no longer exist and the radiolytic products are uniformly distributed throughout the bulk solution.

Under ordinary irradiation conditions—i.e., in the absence of dose rate effects [2,64]—the concentrations of radiolytic products remain low compared to the background levels of dissolved O_2_, H_2_, and cystamine. As a result, their reactions could be modeled as pseudo-first order within the IRT program.

The IRT program also accounts for the effect of ionic strength on all ion–ion reactions, except for the bimolecular self-recombination of e^−^_aq_, for which no ionic strength dependence has been reported [65]. Rate constants were adjusted using the same procedure described in [48,66].

Diffusion coefficients for the various species involved in the IRT simulations were taken from [58,67]. A value of 5.1 × 10^−5^ cm^2^/s was used for H_2_, and 2.4 × 10^−5^ cm^2^/s for O_2_ in aerated solutions at 25 °C. For solutions containing cystamine, a common value of 2 × 10^−5^ cm^2^/s was applied to cystamine and all of its radiolytic derivatives [48], based on their similar molecular sizes and the absence of specific diffusion data for each species.

Finally, the “direct” effects of ionizing radiation on the solutes—O_2_, H_2_, and cystamine—were neglected. This approximation is justified by their low concentrations in the study (0.25 mM O_2_ and 0–10 mM H_2_ or cystamine) relative to bulk water (~55.5 M).

All calculations were performed by simulating short track segments (typically 50–150 μm) of 300 MeV protons, over which the average LET value obtained in the simulations was approximately 0.3 keV/μm at 25 °C. This LET value agrees well with the data reported by Watt [68] and with the recommendations of ICRU Report 49 [69] for liquid water at a density of 1 g/cm^3^. These model calculations therefore provide “track segment” yields corresponding to a well-defined LET. The number of simulated proton histories (typically 40–100) was selected to ensure minimal statistical uncertainty in the averaged chemical yields while maintaining reasonable computational times.

### 2.2. Effect of Dissolved Oxygen in Water Radiolysis

In air-saturated water at 25 °C (with approximately 0.25 mM dissolved oxygen), O_2_ reacts with e^−^_aq_ and H^•^ atoms as follows [1]:

e^−^_aq_ + O_2_ → O_2_^•−^ *k* = 2.3 × 10^10^ M^−1^ s^−1^ (10)

H^•^ + O_2_ → HO_2_^•^, *k* = 1.3 × 10^10^ M^−1^ s^−1^, (5)

where O_2_^•−^ is in pH-dependent equilibrium with its conjugate acid HO_2_^•^ (see supra). The p*K*_a_ for the HO_2_^•^/O_2_^•−^ pair is 4.8 at 23 °C [17]). Based on this p*K*_a_ value, the Henderson-Hasselbalch equation indicates that O_2_^•−^ is the predominant form of the hydroperoxyl radical in neutral aerated water or in aerobic cellular environments at physiological pH.

The time scale for O_2_ to scavenge e^−^_aq_ or H^•^ atoms in aerated water is about 0.2–0.4 μs, estimated using the reciprocal of the “scavenging power”—the product of a solute’s concentration and its rate constant for reaction with primary radicals [2,70]. This time scale roughly corresponds to the end of track expansion and the onset of homogeneous chemistry during low-LET irradiation.

### 2.3. Modeling Water Radiolysis in the Presence of Cystamine: Reaction Scheme

Cystamine is an organic diamino-disulfide compound with the molecular formula RSSR, where R = NH_2_–CH_2_–CH_2_. It is the disulfide form of cysteamine (RSH), an aminothiol derived from cysteine (HS–CH_2_–CH(NH_2_)–COOH), a key amino acid present in most proteins. Known for its radioprotective properties since the pioneering studies of Bacq and coworkers [24,71,72], cystamine plays a crucial role in mitigating oxidative stress within cells and tissues, thereby protecting against radiation-induced damage.

In our simulations of the radiolysis of aerated water in the presence of cystamine, we used the chemical reaction scheme, rate constants, and diffusion coefficients for reactive species as implemented in our IRT program, based on previous studies of the radiolytic oxidation of ferrous to ferric ions in Fricke–cystamine solutions [24,42,48]. Notably, our model for cystamine’s radiation chemistry accurately reproduced the experimental yields of Fe^3+^ in aqueous ferrous sulfate (Fricke)–cystamine solutions irradiated with cobalt-60 γ-rays or fast electrons, without requiring adjustable parameters [42]. This accuracy was consistent across a wide range of cystamine concentrations, regardless of oxygen presence [42]. The strong agreement between our simulated *G*(Fe^3+^) values and the observed data validates the reliability of this reaction scheme. In this work, the water radiolysis reaction scheme (detailed in Table 14.1 of [4]) was expanded to include 17 additional chemical reactions [24], as listed in Table 1.

## 3. Results

### 3.1. Yields of Reactive Species in the Radiolysis of Aqueous Solutions with and Without Added H_2_ Under Deaerated Conditions

**Figure 1:**
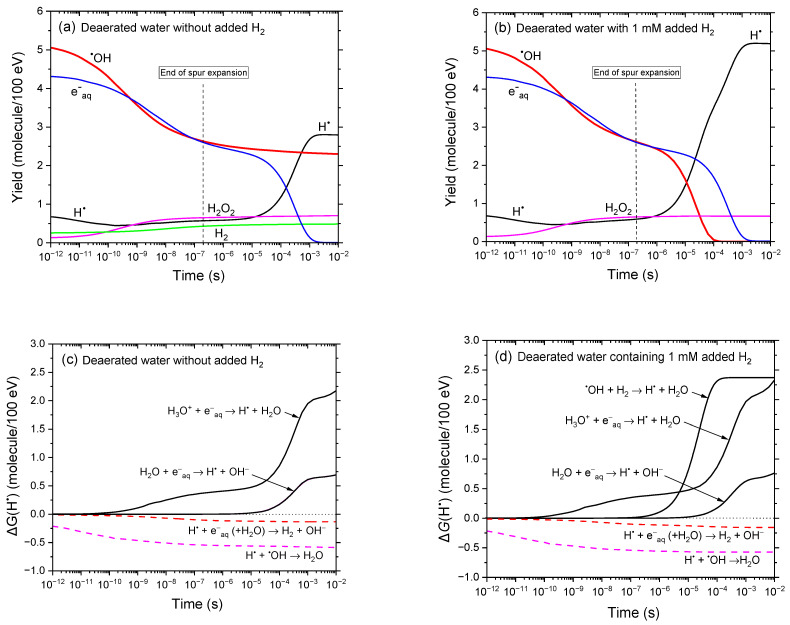
Time evolution of the yields of key reactive species (e^−^_aq_, ^•^OH, H^•^, H_2_O_2_, and H_2_) obtained from our IONLYS-IRT Monte Carlo track chemistry simulations of the radiolysis of deaerated water by 300 MeV protons (LET~0.3 keV/μm) at 25 °C, over a time span from ~1 ps to 10 ms. Panels (**a**,**b**) show the results in the absence and presence of 1 mM added H_2_, respectively. For reference, the vertical dashed line at ~0.2 μs indicates the transition from nonhomogeneous spur kinetics to homogeneous kinetics in the bulk solution [53]. Panels (**c**,**d**) display the time-dependent contributions ∆*G*(H^•^) of individual reactions to the formation (solid black lines) and decay (dashed red and magenta lines) of H^•^ atoms in the absence and presence of added H_2_, respectively, as calculated from the same Monte Carlo simulations (see text).

Figure 1a,b compares the time evolution of various radiolytic yields obtained from our simulations of the radiolysis of deaerated, neutral water by 300 MeV protons over the range from 1 ps to 10 ms, in the absence and presence of 1 mM added H_2_, respectively. The results clearly show that the addition of H_2_ significantly alters the yields of ^•^OH and H^•^, while the yields of the other radiolytic products remain essentially unchanged.

A pronounced decrease—by approximately 2.5 *G*-units—in *G*(^•^OH) is observed in the 1–100 μs time range in the presence of added H_2_ (Figure 1b), consistent with reaction (4), which highlights the strong ^•^OH-scavenging capacity of molecular hydrogen. *G*(^•^OH) steadily declines and approaches zero by ~100 μs. In contrast, the radiolysis of deaerated water—whether or not H_2_ is present—leads to the formation of H^•^ radicals. However, as shown in Figure 1a,b, *G*(H^•^) increases markedly when H_2_ is added, rising from ~2.8 molecules/100 eV at 10 ms (without H_2_) to ~5.2 molecules/100 eV with 1 mM added H_2_. This behavior is further illustrated in Figure 1c,d, which presents the time-dependent contributions ∆*G*(H^•^) from the individual reactions involved in the formation and decay of H^•^ atoms, as calculated in our simulations for 300 MeV proton irradiation. In the absence of H_2_, the primary sources of H^•^ are the following reactions [4,36]:

H_3_O^+^ + e^−^_aq_ → H^•^ + H_2_O *k* = 2.1 × 10^10^ M^−1^ s^−1^ (26)

and

H_2_O + e^−^_aq_ → H^•^ + OH^−^ *k* = 15.8 M^−1^ s^−1^. (27)

When dissolved H_2_ is present, an additional significant contribution arises from reaction (4), in which ^•^OH is reduced by H_2_ to form H^•^, further amplifying the overall *G*(H^•^) yield.

From a radiobiological perspective, although H^•^ is less reactive and less damaging than ^•^OH, it remains a chemically active radical and is certainly not biologically inert [7,10]. This may have important implications under hypoxic conditions—such as those commonly found in solid tumors—where the absence of oxygen limits further reactions that would otherwise neutralize H^•^ (e.g., see reactions (5) and (6)). In such environments, the accumulation of H^•^ radicals could initiate secondary chemical pathways, including the reduction of biomolecules or interactions with metal ions, potentially affecting therapeutic outcomes. While H_2_ acts as an antioxidant, its radioprotective role in oxygen-deprived tissues may therefore be more complex than simply “eliminating” oxidative stress.

### 3.2. Yields of Reactive Species in the Radiolysis of Aqueous Solutions with and Without Added H_2_ Under Aerated Conditions

**Figure 2:**
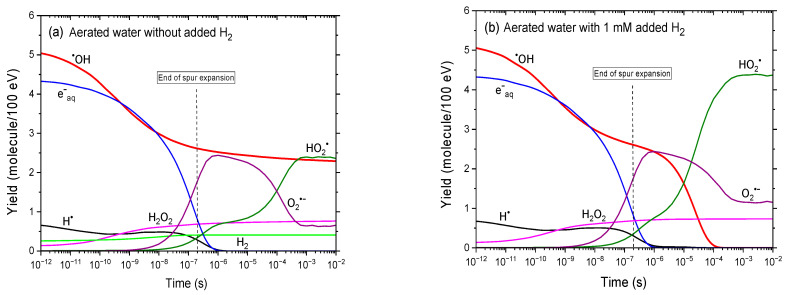

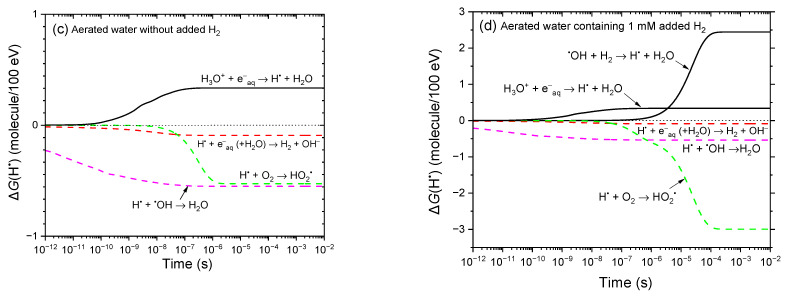
Time evolution of the yields of key reactive species (e^−^_aq_, ^•^OH, H^•^, H_2_O_2_, H_2_, O_2_^•−^, and HO_2_^•^) obtained from our IONLYS-IRT Monte Carlo track chemistry simulations of the radiolysis of aerated water by 300 MeV protons (LET ~ 0.3 keV/μm) at 25 °C, over a time span from ~1 ps to 10 ms. The concentration of dissolved oxygen used in the calculations was 0.25 mM. Panels (**a**,**b**) show the results in the absence and presence of 1 mM added H_2_, respectively. For reference, the vertical dashed line at ~0.2 μs indicates the transition from nonhomogeneous spur kinetics to homogeneous kinetics in the bulk solution [53]. Panels (**c**,**d**) display the time-dependent contributions ∆*G*(H^•^) of individual reactions to the formation (solid black lines) and decay (dashed green, red, and magenta lines) of H^•^ atoms in the absence and presence of added H_2_, as calculated from the same Monte Carlo simulations (see text).

Figure 2a,b compares the time evolution of various radiolytic yields obtained from our simulations of aerated, neutral water irradiated by 300 MeV protons over the range 1 ps–10 ms, in the absence and presence of 1 mM added H_2_, respectively. As in Figure 1a,b, the addition of H_2_ markedly alters the yields of ^•^OH and H^•^. In this case, however, the simultaneous presence of dissolved oxygen further modifies the system by converting e^−^_aq_ and H^•^ into O_2_^•−^ and HO_2_^•^, respectively. These effects are further illustrated in Figure 2c,d, which shows the time-dependent contributions ∆*G*(H^•^) of individual reactions to the formation and decay of H^•^ atoms, in the absence and presence of 1 mM added H_2_, as calculated from our Monte Carlo simulations. For comparison, Figure 1c,d presents the corresponding results obtained in the absence of O_2_.

Let us examine these figures in more detail.

In the absence of added H_2_, the time evolution of *G*(^•^OH) remains essentially unchanged compared to the deaerated case. However, in the presence of 0.25 mM dissolved oxygen, H^•^ atoms are converted into HO_2_^•^ via reaction (5), which proceeds at ~0.3 μs, based on the reciprocal of oxygen’s scavenging power. Additionally, unlike in the radiolysis of deaerated water, hydrated electrons are rapidly scavenged by O_2_ through reaction (10), forming superoxide anion radicals at around 0.2 μs. As a result, most—if not all—e^−^_aq_ are effectively converted into O_2_^•−^, accounting for the sharp rise in *G*(O_2_^•−^) observed between ~10 ns to 1 μs. At longer times, *G*(O_2_^•−^) gradually decreases due to the following reaction [4,36]:

O_2_^•−^ + H_3_O^+^ → HO_2_^•^ + H_2_O *k* = 5 × 10^10^ M^−1^ s^−1^ (28)

This decline continues until *G*(O_2_^•−^) stabilizes at ~0.66 molecule/100 eV around 1 ms. Consequently, *G*(HO_2_^•^) reflects two principal contributions: one from the direct reaction of H^•^ with O_2_ (reaction (5)), and another from the protonation of O_2_^•−^ (reaction (28)). As shown in Figure 2a, *G*(HO_2_^•^) also levels off near 1 ms, reaching ~2.36 molecules/100 eV. The combined yield of HO_2_^•^ and O_2_^•−^ therefore amounts to approximately 3.02 molecules/100 eV in the millisecond time range. For comparison, at the same time point, G(H^•^) reaches ~2.80 molecules/100 eV in the radiolysis of deaerated water without added H_2_ (Figure 1a).

To summarize, in aerated water containing 0.25 mM dissolved O_2_ and no added H_2_, the only oxidizing species remaining at ~1 ms are ^•^OH, HO_2_^•^/O_2_^•−^, and H_2_O_2_, with respective yields of ~2.3, 3.02, and 0.76 molecules/100 eV.

In addition to the effects described above due to the presence of O_2_, the addition of 1 mM H_2_ to aerated water leads to the complete disappearance of ^•^OH radicals after ~100 μs, accompanied by the simultaneous formation of additional H^•^ atoms via reaction (4), followed by HO_2_^•^ formation via reaction (5). As a result, the only radical species remaining in the system at longer times are HO_2_^•^/O_2_^•−^. At ~10 ms, their combined yield reaches ~5.52 molecules/100 eV—a value comparable to the ~5.2 molecules/100 eV observed for *G*(H^•^) in the radiolysis of deaerated water containing 1 mM added H_2_ (Figure 1b). As for H_2_O_2_, its yield remains relatively unchanged, regardless of the presence or absence of O_2_ or H_2_.

As mentioned above, O_2_^•−^ is the predominant form of the hydroperoxyl radical in neutral aerated water at 25 °C or in aerobic cellular environments at physiological pH [10,17]. In other words, radiobiologically, this shift from highly reactive ^•^OH radicals to the less aggressive but longer-lived O_2_^•−^ species may significantly alter the nature of radiation-induced damage, potentially reducing direct oxidative insults to DNA and proteins while favoring slower, redox-driven pathways [7,10]. However, these O_2_^•−^ species should ultimately be neutralized by endogenous antioxidant systems, in particular through the dismutation reaction (6) catalyzed by SOD.

Nonetheless, these considerations suggest that while H_2_ mitigates oxidative stress, its overall radiobiological effects—especially in O_2_-deprived tissues—may be more nuanced than anticipated.

### 3.3. Time Profiles of G(^•^OH) in the Radiolysis of Aerated Water Containing Various H_2_ Concentrations

**Figure 3:**
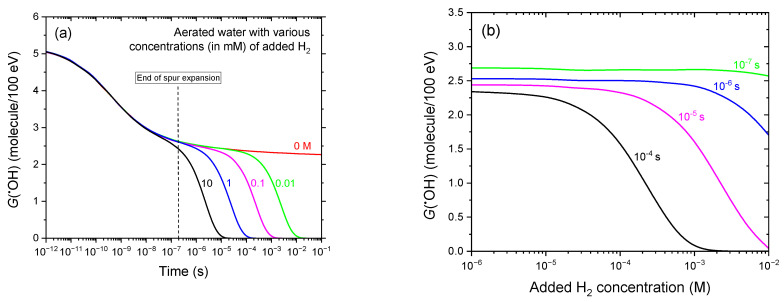
Panel (**a**) shows the time evolution of the ^•^OH radical yield obtained from our IONLYS-IRT Monte Carlo track chemistry simulations of the radiolysis of aerated water by 300 MeV protons (LET~0.3 keV/μm) at 25 °C, over a time span from ~1 ps to 100 ms, in the presence of varying H_2_ concentrations (in mM): 10 (black line), 1 (blue), 0.1 (magenta), and 0.01 (green). The red line shows *G*(^•^OH) in the absence of added molecular hydrogen. The curve for 10 mM H_2_ is included solely for comparison with the corresponding result for 10 mM dissolved cystamine (Figure 4). Note that H_2_ has low solubility in water—~0.78 mM (1.57 mg/L or 1.57 ppm) at standard ambient temperature and pressure (see text). For reference, the vertical dashed line at ~0.2 μs marks the transition from nonhomogeneous spur kinetics to homogeneous kinetics in the bulk solution [53]. Panel (**b**) provides an alternative view of Figure 3a, showing the decrease in *G*(^•^OH) with increasing H_2_ concentration (10^−3^–10 mM) at post-irradiation times of 10^−7^, 10^−6^, 10^−5^, and 10^−4^ s, as obtained from our Monte Carlo simulations. The dissolved oxygen concentration used in the calculations was 0.25 mM.

Figure 3a presents the time-dependent evolution of G(^•^OH) during the radiolysis of aerated water by 300 MeV protons, with added H_2_ concentrations ranging from 0.01 to 10 mM. As shown, increasing the concentration of molecular hydrogen results in a progressively earlier decline in ^•^OH yields. The onset of this decrease—defined as the point where the G(^•^OH) curve begins to deviate from that in the absence of added H_2_—shifts from ~60 μs at 0.01 mM H_2_ to around 30 ns at 10 mM H_2_. This behavior reflects the enhanced efficiency of the scavenging reaction (4), in which ^•^OH radicals are consumed by H_2_ (see supra). At all H_2_ concentrations studied, virtually all radiolytically generated ^•^OH radicals are ultimately eliminated via this reaction.

Specifically, the characteristic time scale for this scavenging process can be estimated from the inverse of the pseudo–first-order rate constant, 1/(*k*[H_2_]), where *k* = 4 × 10^7^ M^−1^ s^−1^. This gives time constants ranging from ~2.5 ms at 0.01 mM H_2_ to ~2.5 μs at 10 mM H_2_. Notably, across the entire H_2_ concentration range studied, ^•^OH removal occurs primarily during the homogeneous chemical stage of radiation action—i.e., after the dissipation of radiation tracks. This observation underscores that the radioprotective effect of H_2_ against ^•^OH radicals manifests predominantly once the initially formed reactive species have diffused and become homogeneously distributed throughout the bulk solution.

Figure 3b provides an alternative representation of Figure 3a, showing how *G*(^•^OH) decreases with increasing H_2_ concentration (10^−3^–10 mM) at post-irradiation times of 0.1, 1, 10, and 100 μs. These results underscore the concentration-dependent scavenging effect of H_2_ on ^•^OH radicals, as revealed by our Monte Carlo simulations.

### 3.4. Comparison of the Antioxidant and Radioprotective Efficiency of H_2_ and Cystamine

**Figure 4:**
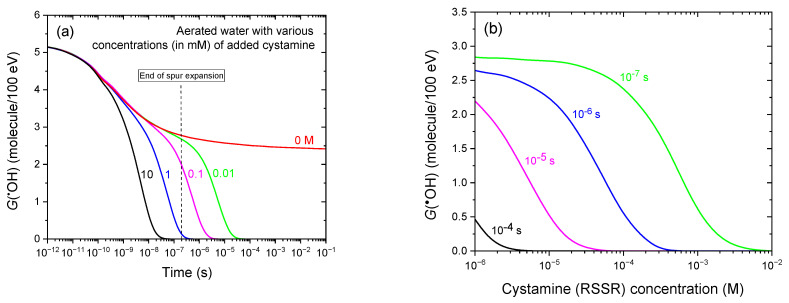
Panel (**a**) shows the time evolution of the yield of ^•^OH radicals as obtained from our IONLYS-IRT Monte Carlo track chemistry simulations of the radiolysis of aerated water by 300 MeV protons (LET ~ 0.3 keV/μm) at 25 °C, over a time range from ~1 ps to 100 ms, in the presence of varying cystamine (RSSR) concentrations (in mM): 10 (black line), 1 (blue), 0.1 (magenta), and 0.01 (green). The red line shows *G*(^•^OH) in the absence of cystamine. For reference, the vertical dashed line at ~0.2 μs marks the transition from nonhomogeneous spur kinetics to homogeneous kinetics in the bulk solution [53]. Panel (**b**) provides an alternative view of Figure 4a, showing the decrease in *G*(^•^OH) with increasing RSSR concentration (10^−3^–10 mM) at post-irradiation times of 10^−7^, 10^−6^, 10^−5^, and 10^−4^ s, as obtained from our Monte Carlo simulations. The concentration of dissolved oxygen used in the simulations was 0.25 mM.

Figure 4a shows the time-dependent evolution of *G*(^•^OH) during the radiolysis of aerated water by 300 MeV protons, under the same conditions as in Figure 3a but with cystamine (RSSR) concentrations ranging from 0.01 to 10 mM. As in Figure 3a,b, Figure 4b provides an alternative representation of Figure 4a, depicting the decrease in *G*(^•^OH) with increasing RSSR concentration (10^−3^–10 mM) at post-irradiation times of 0.1, 1, 10, and 100 μs. These results demonstrate the strong, concentration-dependent scavenging efficiency of cystamine toward ^•^OH radicals, as predicted by our Monte Carlo simulations.

As observed with molecular hydrogen, the addition of cystamine induces a pronounced and progressive reduction in ^•^OH yields due to the scavenging reaction between ^•^OH and RSSR (reaction (13)) [42,75,76]. This decline becomes increasingly steep and occurs earlier in time as the RSSR concentration increases, with the onset of *G*(^•^OH) reduction shifting from ~100 ns at 0.01 mM RSSR to ~100 ps at 10 mM—reflecting the high reactivity of cystamine toward ^•^OH radicals (see, e.g., [24,42,48] and references therein). From a radiation-chemical standpoint, it is worth noting that, across the concentration range studied, ^•^OH removal by RSSR primarily occurs during the nonhomogeneous chemical stage of radiation action—earlier than for H_2_, which acts predominantly at homogeneity.

A direct comparison of the antioxidant efficiency of H_2_ and cystamine at an identical concentration of 10 mM further underscores this difference. In the presence of RSSR, *G*(^•^OH) drops sharply from ~4.4 molecules/100 eV at ~100 ps to nearly zero by ~30 ns. In contrast, with H_2_, the ^•^OH yield begins to decline later (~2.8 molecules/100 eV at ~30 ns) and reaches zero only at ~15 μs. These results clearly demonstrate that cystamine is markedly more effective than H_2_ in scavenging ^•^OH radicals: it reacts faster and eliminates ^•^OH much earlier—even at lower concentrations—highlighting its superior antioxidant efficiency and radioprotective potential.

Despite its somewhat lower scavenging efficiency compared to cystamine, H_2_ offers a distinct advantage: its non-toxic nature. While cystamine is highly effective in eliminating ^•^OH radicals, its potential cytotoxicity limits its broader application—especially in biomedical and therapeutic settings where safety is paramount. In contrast, H_2_ combines appreciable antioxidant properties with an excellent safety profile, posing no harm to cellular structures or functions during its scavenging activity [45].

## 4. Discussion, Conclusions, and Perspectives

Our Monte Carlo track chemistry simulations quantitatively demonstrate that molecular hydrogen significantly reduces the yield of hydroxyl radicals following low-LET irradiation, without noticeably altering the yields of other radiolytic species under conditions mimicking cellular environments. This selective scavenging of ^•^OH occurs increasingly rapidly with rising H_2_ concentrations. Notably, even at concentrations as low as 0.01 mM—well below the ~0.3–0.78 mM range typically used in experimental or clinical studies—H_2_ effectively eliminates nearly all ^•^OH radicals. However, when benchmarked against cystamine, whose sulfur-based radical-quenching capability is well established, H_2_ proves less efficient in neutralizing reactive oxygen species (ROS). Cystamine exhibits markedly higher scavenging efficacy, achieving complete ^•^OH elimination at earlier times and at lower concentrations. From a radiation-chemical standpoint, cystamine’s reactivity translates into superior radioprotective performance.

Yet, this enhanced chemical efficacy comes with important trade-offs. Cystamine is known to exert cytotoxic effects at elevated concentrations, limiting its suitability for clinical applications. In contrast, H_2_ offers a key advantage—biological safety—consistent with experimental findings. It is non-toxic, chemically inert under physiological conditions, and readily permeates cellular membranes. These properties enable H_2_ to exert antioxidant effects without interfering with normal cellular functions, making it particularly appealing for medical use in sensitive contexts, such as protecting healthy tissues during radiotherapy or managing oxidative stress-related diseases.

Beyond direct radical scavenging, additional mechanisms—such as the modulation of oxidative stress signaling pathways, anti-inflammatory effects, and stimulation of cellular repair processes (see, e.g., [77])—may also underline the radioprotective action of H_2_. These complementary biological effects reinforce its potential as a therapeutic antioxidant. Taken together, the unique combination of selectivity, efficacy, and non-toxicity positions molecular hydrogen as a particularly promising candidate for antioxidant-based interventions, including the protection of healthy tissues during cancer radiotherapy and the mitigation of oxidative stress in diverse pathological conditions.

Importantly, our simulations also raise a cautionary consideration: although H_2_ efficiently scavenges ^•^OH radicals, it concurrently leads to the formation of H^•^ atoms—species that, while less reactive than ^•^OH, are not biologically inert. In oxygen-deficient environments, such as hypoxic tumor tissues, where oxidative pathways for detoxifying H^•^ are limited or inactive, these radicals may accumulate and engage in alternative chemical reactions. These could include the reduction of biomolecules or interactions with transition metal ions, potentially influencing cellular redox balance or therapeutic outcomes. Therefore, the radiobiological effects of H_2_—particularly under hypoxic conditions—may be more nuanced than previously anticipated. Notably, the balance between ^•^OH elimination and H^•^ formation should be carefully considered when evaluating the use of hydrogen-rich water in radiobiology and radiation therapy.

In summary, molecular hydrogen offers a compelling balance of efficacy and safety as a selective antioxidant and radioprotector. Although it may not equal the chemical potency of cystamine, its exceptional biocompatibility and ability to selectively scavenge the most cytotoxic ROS position it as a highly attractive candidate for further investigation in both fundamental and clinical radiobiology.

With respect to the radiolysis of deaerated or aerated water in the presence of H_2_, we were unable to identify experimental yield values for the various radiolytic species as a function of time after irradiation that could serve as a direct benchmark for our results. The availability of such data would be highly valuable, as they are essential for fully validating simulations under these specific conditions.

Finally, two promising directions for future work emerge.

First, our model could be extended to high-LET irradiation by lowering the proton energy from 300 MeV to, for example, 150 keV, thereby covering the LET range from ~0.3 to 72.2 keV/μm [48]. Higher LET is expected to increase local ^•^OH radical concentrations within tracks, enhancing intra-track recombination and reforming water or producing hydrogen peroxide. As a result, the scavenging efficiency of H_2_ toward ^•^OH may decrease due to competing (^•^OH + H^•^) and (^•^OH + ^•^OH) reactions.

Second, the effect of elevated dose rates, particularly in the context of the ‘FLASH effect’ in radiotherapy, also warrants investigation. As with higher LET, increased dose rates raise ^•^OH concentrations in the bulk solution, promoting inter-track recombination into H_2_O and H_2_O_2_, which may again reduce the net ^•^OH scavenging by H_2_.

Both avenues are currently under active study in our laboratory and should provide new insights into the interplay between radiation quality (LET), dose delivery, and the antioxidant and radioprotective action of molecular hydrogen.

## Figures and Tables

**Table 1:** Reaction scheme used in our simulations of the radiolysis of aerated water–cystamine solutions to model the radiation chemistry of cystamine (RSSR) ^1,2^.

Reactions	*k* (M^−1^ s^−1^)	Reaction No.
RSSR + e^−^_aq_ → (RSSR)^•−^	4.1 × 10^10^	(11)
RSSR + H^•^ → RS^•^ + RSH	8 × 10^9^	(12)
RSSR + ^•^OH → (RSSR)^•+^ + OH^−^	1.7 × 10^10^	(13)
(RSSR)^•−^ + H^+^ → RS^•^ + RSH	4.2 × 10^9^	(14)
2(RSSR)^•+^ → (RSSR)^2+^ + RSSR	2.5 × 10^9^	(15)
RS^•^ + RSSR → RSSSR + R^•^	10^6^	(16)
RSH + e^−^_aq_ → R^•^ + HS^−^	3 × 10^10^	(17)
RSH + H^•^ → RS^•^ + H_2_	1.8 × 10^9^	(18)
RSH + ^•^OH → RS^•^ + H_2_O	1.7 × 10^10^	(19)
RS^•^ + RSH → (RSSR)^•−^ + H^+^	3.5 × 10^8^	(20)
R^•^ + RSH → RH + RS^•^	1.1 × 10^8^	(21)
RS^•^ + RS^•^ → RSSR	1.5 × 10^9^	(22)
RS^•^ + O_2_ → RSOO^•^	2 × 10^9^	(23)
RSOO^•^ + RSH → RSO^•^ + RSOH	2 × 10^6^	(24)
RH + ^•^OH → R^•^ + H_2_O	5 × 10^8^	(2)
R^•^ + O_2_ → ROO^•^	2 × 10^9^	(3)
(RSSR)^•−^ + O_2_ → RSSR + O_2_^•−^	5.1 × 10^8^	(25)

^1^ Below pH 8, cystamine predominantly exists in the form of the double protonated molecule ^+^NH_3_–CH_2_–CH_2_–S–S–CH_2_–CH_2_–NH_3_^+^ (p*K*_a_~8.7–9 for both of the –NH_3_^+^ groups) [48,73,74]. ^2^ The rate constants (*k*) quoted here for reactions between ions are in the limit of infinite dilution (i.e., not corrected for the effects due to the ionic strength of the solutions).

## Data Availability

Data generated or analyzed during this study are provided in full within the article. For further inquiries, please contact the authors directly.
